# News and Coming Events

**Published:** 1907-10-12

**Authors:** 


### NEWS AND COMING EVENTS.

### THE LONDON MEDICAL EXHIBITION.

This exhibition, being the third of the series organised by *The British and Colonial Druggist*, was held at the Royal Horticultural Hall, Vincent Square, Westminster, from October 7 to October 11, and can be pronounced a further successful advance upon the two previous undertakings. The aim of the organisers is to cater exclusively for the medical profession, and their efforts this year have been rewarded with recognition far in excess of any previous experience, the attendance being larger and more medically representative than before. In the extensive array of exhibits in drugs, pharmaceutical preparations, prepared foods, surgical dressings and appliances, and invalid furniture, many new and highly interesting features were in evidence. Space will not admit of a description of all the exhibits, but some of the most important may be mentioned with advantage. Messrs. Duncan, Flockhart and Co. had an extensive display of chloroform, ether purificatus, and ethyl chloride, but particular attention was drawn to glyceroformate, a preparation indicated in neurasthenia, boraldehyde (boric acid and formaldehyde), a most active antiseptic for septic throats, etc.; a preparation of collodion and ether was specially recommended as being more effective in its action than belladonna. Mr. William Martindale had on view his latest Pharmacopœia, which has been very highly reported upon by the leading medical journals and is an invaluable reference book for the busy practitioner. The closest interest was shown in the high-class medical preparations on this stand, prominent among which were Faexin, a pure yeast in powder for application in boils, furuncles, and skin eruptions; Maltoferrose, sufficiently indicated by its name; and Maricol (*Magnesii Ricinoleas*), suitable for children. We recently called attention in our columns to a plant called *Combretum Sundaicum*, which had been accidentally discovered to be an effective remedy for the opium habit, but had not succeeded in inducing investigations in the laboratory by chemists in this country. Mr. Martindale had, however, a specimen of this plant on view, which gives promise of so useful a preparation being available as soon as a convenient form of administration is determined on and the proper dosage ascertained by clinical test. Messrs. C. J. Hewlett and Son exhibited their pepsin and bismuth preparations, which are held in much high repute, and some antiseptic preparations, including cream in collapsible tubes for skin affections, and "Creosalgen," an antiseptic germicide disinfectant so powerful as to bear dilution of 1 to 100 parts of water to make an effective disinfectant. Equipoise, Ltd., had on view the entire range of their furniture, including beds, couches, lounges, easy chairs, and even bath chairs. The distinguishing feature of the invalid furniture bearing the name of "Equipoise" is that every article is capable of adjustment to any position by mechanical action which can be worked by the patients or attendant.

---

The Transatlantic tide of travel from Southampton and other Channel ports and from Liverpool to New York is still at its height, and will hardly have ceased before the rush from New York to the Mediterranean will have set in. To meet this ever-increasing trade the White Star Line have organised a regular service of their enormous ships, headed by the *Cedric* (21,035 tons, the largest ship in Mediterranean waters). Other vessels on this Mediterranean line are the twin-screw monsters *Cretic, Canopic, Romanic*, and *Republic*. These vessels touch at the Azores, a passage about six days from New York, and thence call at Gibraltar and Naples, the terminal port being Genoa. Other extensions of the White Star Mediterranean service during the coming winter will include a call at Madeira and also an extension in some cases to Alexandria.

The following Entrance Scholarships have been awarded to-day at the London Hospital Medical College :—Price scholarship in science, value £120, Mr. T. D. Williams; science scholarships, value £60, Mr. N. R. Rawson; science scholarships, value £35, Mr. J. H. Lloyd; Price scholarship in anatomy and physiology, Mr. G. J. F. Jessel (University of Oxford); Epsom scholarship, value £126, Mr. E. H. Henson.

---

During the summer considerable alterations have been in hand at the London Hospital Medical College. An inoculation department was started two years ago. Up to the present the work of this department has been carried out in the General Bacteriological Laboratory. An extension of this laboratory has now been effected so that the inoculation department will be worked, independently of the routine bacteriology, under the control of the bacteriologist to the hospital. In the new department provision has been made both for teaching and research.

---

Guy's Hospital Medical School.—The following Entrance Scholarships and certificates have been awarded :—Senior Science Scholarships for University Students, £50, J. G. Saner, Caius College, Cambridge. Junior Science Scholarships, £150, J. F. Mackenzie; £60, R. D. Passey. Certificates : K. J. Bennett, G. E. Genge-Andrews, S. Keith. Entrance Scholarships in Arts : £100, C. S. L. Roberts, Cheltenham College; £25, G. D. Eccles, Plymouth Technical School, and £25, G. F. Romer, Malvern College, equal.

---

In connection with the Asylum of the Mission to Lepers in India, at Sabathu, in the Punjab, there is a ward for English lepers. The rooms open off a verandah, on which the inmates can sit and enjoy the air. Erected for only three inmates, the house is now quite overcrowded, and the sitting-room has had to be utilised as a bedroom. An extension of the European part of the Asylum is much needed. The Organising Secretary of the Mission is Mr. John Jackson, F.R.G.S., 33 Henrietta Street, Strand, London, W.C.

---

A Provincial Sessional Meeting of the Royal Sanitary Institute will be held at the Council Chamber, Town Hall, Ipswich, on Saturday, October 19, at 11 a.m., when a discussion will take place on "The Recent Report of the Royal Commission on Tuberculosis." The chair will be taken at 3 p.m, by Colonel J. Lane Notter, M.A., M.D., R.A.M.C. Tickets for admission of visitors to the sessional meeting may be had on application to Dr. A. M. N. Pringle, Medical Officer of Health, Exchange Chambers, Ipswich, who is kindly acting as the local hon. secretary of the meeting.

The opening of the new session at University College Hospital corresponds with the opening of a new era in the history of the Medical School. The school will begin its work in the magnificent new buildings provided for it by the generosity of Sir Donald Currie. These buildings provide accommodation for the departments of medicine and clinical medicine, surgery and clinical surgery, midwifery and gynæcology, pathology including morbid anatomy, clinical pathology and bacteriology, forensic medicine, mental physiology and mental diseases, dental surgery, practical pharmacy, and other departments for the study of special diseases such as those of the eye, skin, ear, and throat, and for instruction in the use of anæsthetics, and in electro-therapeutics and the application of the *x*-rays. Each of the departments is also equipped for more advanced work and provides facilities for research. Very extensive research laboratories and special research rooms in pathology have been set aside.

